# Procyanidins and Their Therapeutic Potential against Oral Diseases

**DOI:** 10.3390/molecules27092932

**Published:** 2022-05-04

**Authors:** Huan Chen, Wanyu Wang, Shiyang Yu, Huimin Wang, Zilu Tian, Song Zhu

**Affiliations:** 1 Department of Prosthodontics, School and Hospital of Stomatology, Jilin University, Changchun 130012, China; chenhuan_9409@163.com (H.C.); onepiece_1991@126.com (S.Y.); hmwang21@mails.jlu.edu.cn (H.W.); tianzlchongchong@163.com (Z.T.); 2 Department of Pharmacy, Union Hospital, Tongji Medical College, Huazhong University of Science and Technology, Wuhan 430022, China; wanyu15810@163.com

**Keywords:** procyanidins, oral disease, bioavailability, drug delivery, antioxidation

## Abstract

Procyanidins, as a kind of dietary flavonoid, have excellent pharmacological properties, such as antioxidant, antibacterial, anti-inflammatory and anti-tumor properties, and so they can be used to treat various diseases, including Alzheimer’s disease, diabetes, rheumatoid arthritis, tumors, and obesity. Given the low bioavailability of procyanidins, great efforts have been made in drug delivery systems to address their limited use. Nowadays, the heavy burden of oral diseases such as dental caries, periodontitis, endodontic infections, etc., and their consequences on the patients’ quality of life indicate a strong need for developing effective therapies. Recent years, plenty of efforts are being made to develop more effective treatments. Therefore, this review summarized the latest researches on versatile effects and enhanced bioavailability of procyanidins resulting from innovative drug delivery systems, particularly focused on its potential against oral diseases.

## 1. Introduction

Procyanidins (PCs), also called proanthocyanidins and condensed tannins, are widely found in flowers, nuts, fruits, bark, and seeds of various plants [1,2]. PCs with a degree of polymerization of 2–4 or more are called oligomeric procyanidins (OPCs) and polymeric procyanidins (PPCs), respectively [3]. In most cases, the flavane-3-alcohol unit is a substituted derivative of catechin (C), epicatechin (EC), or its C4–C8 or C6 bond (type B) [4]. Recently, it has been showed PCs has predominant pharmaceutical values due to its antioxidant [5], antibacterial [6], anti-inflammatory [7], antineoplastic [8], anti-allergic [9], lipid-lowering, and anti-obesity properties [10]. As a result of these properties, they have been widely recognized and applied in the healthcare industry [11].

Oral health problems, particularly periodontal disease, dental caries, and endodontic root canal infections, are among the most damaging processes in the mouth and a costly burden on the global public [12].According to a review released by the World Health Organization (WHO), oral diseases remain a global problem despite significant progress in developing oral health in some countries [13]. The conventional oral diseases including periodontitis and dental caries are regarded as the infectious diseases, since they are initiated by plaque biofilm formation [14]. In addition, periodontitis leads to alveolar bone destruction and subsequent tooth loss, and develops due to pro-inflammatory cytokine production induced by periodontopathic bacteria [15]. Besides, recurrent aphthous ulcer is characterized by prolonged lesion with severe pain, which may induce from an overload of reactive oxygen species (ROS), occurring in up to 20% of cases [16]. Oral cancers, especially oral squamous cell carcinoma (OSCC) are the main cause of oral disease death, which are partly due to an imbalance between cell growth capacity and elimination mechanisms [17]. Thus, it is necessary to pay great attention to the treatment of oral diseases to maintain oral health and improve the quality of life.

Previous studies have shown that PCs possessed capability to reduce the excessive expression of inflammatory cytokines and inhibit production of inflammation-producing enzymes [18]. In addition, PCs can cause the disruption of bacterial membrane and lead to the leakage of intracellular substances, exerting an antibacterial activity [19]. In general, PCs exert anti-inflammatory effects by regulating nuclear factor erythroid 2-related factor 2 (Nrf2), the nuclear factor-κB (NF-κB) and mitogen-activated protein kinase (MAPK) pathways, as well as inhibiting ROS production and decreasing mitochondrial membrane potential [20,21,22,23,24,25,26,27,28]. Besides, the antineoplastic effects of PCs are achieved through regulating autophagy, apoptosis, epithelial-to-mesenchymal transition (EMT) transformation, drug resistance, tumor stem cell renewal and proliferation of tumor cells [29,30,31,32,33,34,35].Moreover, PCs also have excellent pharmacological effects in the therapy of Alzheimer’s disease [36], diabetes [37], rheumatoid arthritis and obesity [38]. Thus, PCs are considered as a kind of promising candidates to use as potent pharmaceutical for disease prevention and therapeutics.

**Figure 1:**
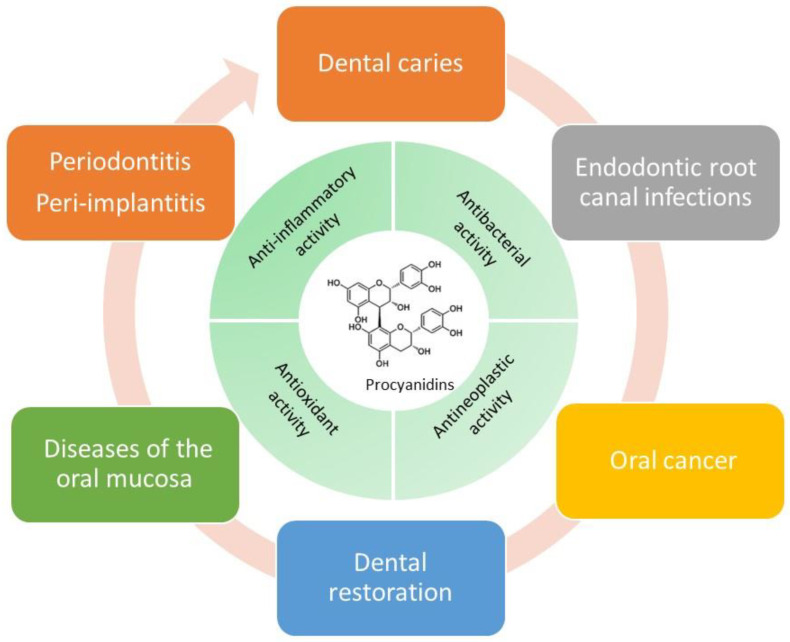
Structure, properties and therapeutic applications on oral diseases of procyanidins. Notes: Procyanidins, as an emerging pharmaceutical with multifunction, have versatile properties such as antioxidant activity, antibacterial activity, anti-inflammatory activity and antineoplastic activity. It has the capacity to therapy diverse oral diseases including oral cancer, periodontitis, dental caries, diseases of the oral mucosa, endodontic root canal; peri-implantitis and dental restoration.

Nevertheless, with respective clinical application, the proven benefits of PCs have some stability and bioavailability issues. Therefore, extensive efforts have been made to increase the bioavailability of PCs by encapsulating it in drug delivery systems including poly(lactic-co-glycolic acid) (PLGA) and poly(D,L-lactic acid) (PLA) nanoparticles [39,40], polysaccharide-based nanoparticles [41,42,43,44,45], protein-based nanoparticles [46,47], modified hydroxyapatite inorganic nanoparticles [48], metal nanoparticles [49,50,51,52,53], ultradeformable liposomes (PUDL) and solid lipid nanoparticles (SLN) [54,55]. Significant advances in bioavailability-related fields guarantee the enhanced pharmaceutical efficacy and further clinical value of PCs as an emerging versatile drug. As shown in Figure 1, The present article aims to summarize the current state of PCs application in oral diseases treatment.

## 2. Pharmacological Mechanism of Procyanidins

### 2.1. Antioxidant Activity

Oxidative stress is defined as the imbalance between the production and elimination of ROS, and oxidative stress caused by excess ROS can cause oxidative damage to cells [56]. Former Studies have demonstrated that PCs can exert antioxidant effects by inhibiting enzyme activity, eliminating free radicals, and resisting oxidative stress [57,58,59].

Studies have shown that PCs have a good scavenging effect on ROS by scavenging free radicals such as O^2−^ and NO^−^ [52]. In addition to serve as a prominent antioxidant agent for radical scavenging, Jimenez-aspee et al. demonstrated that PCs in the pulp of Cortex pedunculata can inhibit the activities of lipoxygenase and enzymes involved in the enzyme peroxidation [57]. Moreover, Bak et al. suggest that PCs from wild grape seed effectively inhibit the production of oxidative mediators, which achieved by preventing the activation of NF-κB and p38 pathways [58].

### 2.2. Antibacterial Effect

Infectious diseases caused by bacteria constitute the main cause of morbidity and mortality throughout the world and mainly in developing countries [60]. So far, the mechanisms of PCs antibacterial activity mainly include: 1. Inhibition of bacterial adhesion and biofilm formation, 2. Destroy the integrity of cell membrane/wall, 3. Inhibit extracellular microbial enzymes and deprive microbial substrates needed for growth [61].

Philips et al. noted that the components of cranberry, particularly PCs, can interfere with the bacterial adhesion stage, reducing biofilm formation and/or reducing inflammation to fight pathogens [62]. Several clinical trials have shown that cranberry procyanidins have anti-adhesion properties and are important in preventing recurrent urinary tract infections [63]. Kim et al. demonstrated that PCs inhibit bacterial adhesion by restraining *Streptococcus mutans* derived glucose transferase (GTF) and extracellular polysaccharides (EPS) without killing the organism [64]. Lacombe et al. believed that PCs play an antibacterial role probably since they have metal ion chelation effect similar to EDTA, which can bind Ca^2+^ and Mg^2+^ on the membrane to destabilize cell membrane, release lipopolysaccharide (LPS), and increase cell membrane permeability [65]. Tamura et al. found that PCs trimers have antibacterial effects on foodborne bacteria, especially *Bacillus cereus* (*B. cereus*), the mechanism is that PCs trimer present in the peanut skin disrupts cell wall integrity of *B. cereus* [66]. Notably, PCs in cranberries have been shown to inhibit the activity of microbial F-ATPase, thus making bacterial survival extremely difficult [67,68].

### 2.3. Anti-Inflammatory Activity

Inflammation is the host’s defensive response to tissue damage or infection caused by a variety of stimulus, such as chemicals, physical trauma, and infectious agents [69].

PCs exert anti-inflammatory activity by regulating MAPK, Nrf2, NF-κB signaling pathway, mitochondrial membrane potential and calcium channel. Nrf2 is an important transcription factor that benefits to cell survival during oxidative stress [20,21]. Ubiquitin binding protein p62 is important for selective autophagy and antioxidant responses [22]. It has recently been found that p62 protects cells from oxidative stress by activating Nrf2 [23]. Lu et al. demonstrated that procyanidins B2 (PCB2) enhances the system of antioxidant via adenosine monophosphate-activated protein kinase (AMPK)/Nrf2/p62 signaling axis, specifically speaking, PCB2 treatment increase p-AMPK levels, along with the letilation of Nrf2 and gather Nrf2 to nucleus, which can promote the expression of NAD(P)H quinone oxidoreductase 1 (NQO1), heme oxygenase-1 (HO-1), and γ-glutamylcysteine synthetase (γ-GCS). Besides, Nrf2 activate by PCB2 up-regulated the expression p62, and p62 stimulate the activation of Nrf2 in turn, which exert a positive loop between Nrf2 and p62 [70]. NF-κB transcription factor plays a key role in the regulation of inflammatory responses [71]. Procyanidins A2 (PCA2) inhibits the release of Interleukin-6 (IL-6), tumor necrosis factor-α (TNF-α), prostaglandin E2 (PGE2), and nitric oxide (NO), through the IκB/NF-κB P65 pathway, thus exerting anti-inflammatory effect [10].

One study demonstrated that procyanidins A1 (PCA1) inhibit LPS-induced oxidative stress through IκB/NF-κB p65 signaling. Meanwhile, PCA1 inhibits the production of intracellular ROS in vitro and reduces the depletion of mitochondrial membrane potential [27]. NOD-like receptor family, pyrin domain containing 3 (NLRP3) inflammasome is a cytoplasmic protein complex, and mediates inflammatory respond by depending on the release of Cathepsin B [28,72]. Procyanidin B2 (PCB2) inhibits monosodiumurate (MSU)-induced inflammatory response in gout by inhibiting the NLRP3 inflammasome pathway, thereby reducing Interleukin-1β (Il-1β) and cathepsin B release [72].

**Figure 2:**
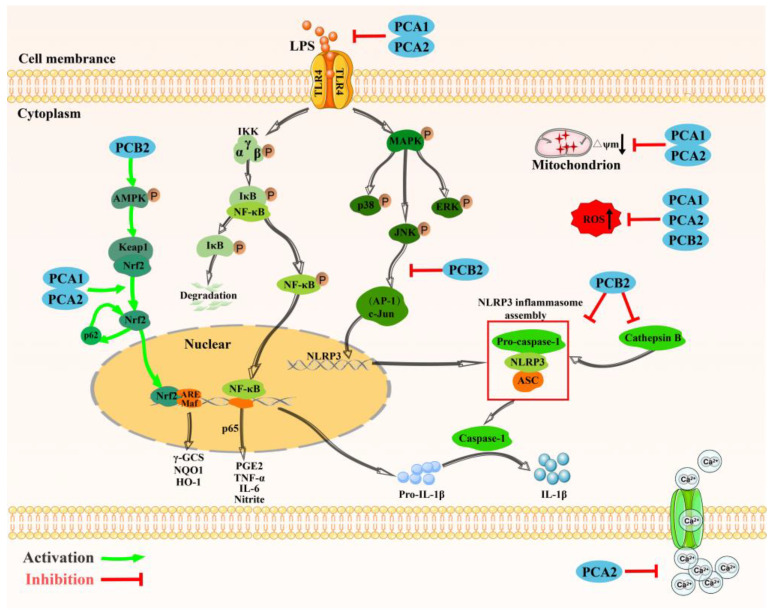
The anti-inflammation signal pathways of procyanidins. Notes: (1). Procyanidins activated the AMPK/Nrf2 pathway; targeted its downstream gene contributing to the increased level of antioxidant genes NQO1, HO-1 and γ-GCS. (2). Procyanidins inhibits the release of inflammatory factors IL-6, TNF-α, PGE2, NO through the IκB/NF-κB p65 pathway (Note: PCA1 show no effect of PEG2). (3). Procyanidins inhibit LPS activated AP-1/c-Jun pathway decreasing the gene expression of NLRP3, and suppress subsequent caspase-1 activation and the release of IL-1β. (4). Besides, Procyanidins can also lower the production of ROS, reverse decreased mitochondrial membrane potential, inhibit Ca^2+^ exclusion. Abbreviation: PCA1, procyanidins A1; PCA2, procyanidins A2; PCB2, procyanidins B2; AMPK, adenosine monophosphate-activated protein kinase; Nrf2, nuclear factor erythroid 2-related factor 2; NQO1, NAD(P)H quinone oxidoreductase 1; HO-1, heme oxygenase-1; γ-GCS, γ-glutamylcysteine synthetase; LPS, lipopolysaccharide; MAPK, mitogen-activated protein kinase; IKK, inhibitor of kappaB kinase; IκB, nuclear factor kappa-B; NF-κB, nuclear factor kappa-B; PGE2, prostaglandin E2; TNF-α, tumor necrosis factor-α; IL-6, Interleukin-6; JNK, c-Jun N-terminal kinases; ERK, extracellular signal-regulated kinase; NLRP3, NOD-like receptor family pyrin domain containing 3; IL-1β, Interleukin-1β; ROS, reactive oxygen species.

MAPKs family members mainly include extracellular signal-regulated kinase (ERK), stress-activated protein kinase (JNK) and P38 MAPK. PCB2 inhibit LPS activated activator protein-1 (AP-1)/c-Jun pathway increasing the gene expression of NLRP3, and suppress subsequent caspase-1 activation and IL-1β secretion in endothelial cells. Besides, PCB2 also lower LPS induced the production of ROS in endothelial cells [18]. All pathways mentioned in this section are concluded in detail in Figure 2.

### 2.4. Antineoplastic Activity

According to 2020 estimates from WHO, cancer is the leading cause of death in countries around the world, which is characterized by abnormal and uncontrolled cell growth [73,74]. Tumor growth underlies all aspects of cancer development. Therefore, inhibition of tumor cell proliferation is considered as a promising cancer treatment strategy [75]. PCs have been found to have significant antitumor effects in breast cancer, lung cancer, hemangioma, colon cancer and other cancers [41,76,77,78,79,80].

There is much evidences suggest that the activation of Nrf2 would lead to cancer cell proliferation and chemotherapy resistance and is related with poor prognosis of patients [29]. Studies have shown that PCs treatment can rapidly accelerate the degradation of activated Nrf2 expression by activating cysteine proteases in nucleus through phosphorylated insulin-like growth factor 1 receptor (IGF-1R), which can inhibit the proliferation of A549 cells [30].

Cancer stem or initiating cells (CSCs) is a rare population of undifferentiated cells, plenty of evidence shows that CSCs is closely associated with drug resistance, carcinoma recurrence and tumor metastasis [31]. A study found that both procyanidin B2 3,3″-di-O-gallate (B2G2) and grape seed procyanidins (GSP) can inhibit the self-renewal of CSCs via inhibiting Notch1 pathway activated by Jagged1 (Notch1 ligand) and restraining the transcription of Notch1 regulatory target gene HES-1, which contributes to the control of proliferating prostate cancer cells [32].

Multidrug resistance (MDR) is one of the dominant reasons of chemotherapy failure in cancer patients, and the expression of p-glycoprotein (P-gp) has close relation with MDR, which is responsible for pumping therapeutic drugs out of cancer cells [33]. Zhao et al. found GSP can significantly decrease the expression of P-gp via inhibiting the nuclear translocation of Y-box binding protein 1 (YB-1) from cytoplasm through MAPK/ERK pathway by reducing the phosphorylation of ERK1/2. Besides, GSP can also block the generation of P-gp by down-regulating NF-κB/p65 activity, eventually leading to reversal MDR. However, authors mentioned that the new mechanism is limited to human ovarian cancer cells of A2780/T and A2780 in vitro. Therefore, it is worth further exploring if GSP can reverse MDR in other cancer cells [34].

Epithelial-to-mesenchymal transition (EMT) is a process of cancer cell in which tumor cells lose their epithelial properties and turn into spindle morphology. And EMT is strongly connected with tumor metastasis. Procyanidin C1 (PC1) from the Cinnamomi Cortex extract can inhibit the process of EMT through restraining TGF-β induced phosphorylation of Smad-2, and further down-regulated the snail, E-cadherin and fibronectin in A549 cells [35].

**Figure 3:**
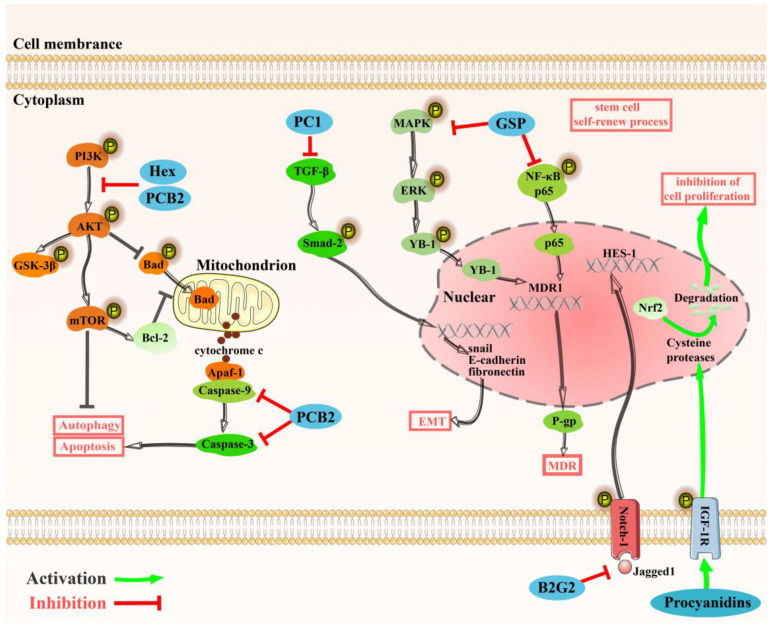
The antineoplastic mechanism of procyanidins. Notes: (1). Procyanidins can inhibit cell proliferation by phosphorylated IGF-1R, which can activate cysteine proteases and accelerate the degradation of activated Nrf2. (2). B2G2 can inhibit stem cell self-renew process through inhibiting the activitation of Notch1 pathway. (3). GSP can reverse MDR by suppressing both MAPK/ERK/YB-1 and NF-κB p65 pathway. (4). PC1 inhibits EMT by inhibiting TGF-β-induced phosphorylation of Smad-2, and further down-regulates Snail, E-cadherin and fibronectin. (5). HEX can inhibit PI3K/Akt/GSK-3β and PI3K/Akt/Bad signaling pathways, and induce the mitochondrial apoptosis pathway of cancer cells. In addition, PCB2 also plays an anti-proliferative role and stimulates apoptosis, induces autophagy of cancer cells through the Akt/mTOR pathway. Abbreviation: IGF-1R, insulin-like growth factor 1 receptor; B2G2, B2 3;3″-di-O-gallate; MDR, multidrug resistance; GSP, grape seed procyanidins; P-gp, p-glycoprotein; YB-1, Y-box binding protein 1; EMT, Epithelial-to-mesenchymal transition; PC1, procyanidin C1; Hex, hexamer; Bcl-2, B-cell lymphoma-2.

In addition, the hexamer (Hex) which is one of trimer procyanidins, induced apoptotic cell death through the mitochondrial pathway and is involved in autophagy by upregulating genes in colorectal cancer cells (Caco-2 cells). Mechanistically, Hex inhibited both PI3K/Akt/GSK-3β and PI3K/Akt/Bad signaling pathway, increasing the translocation of Bad to the mitochondria and cytochrome c to cytoplasm, finally induced mitochondrial apoptosis pathway of cancer cell. Moreover, Hex also blocked the Caco-2 cell cycle at G2/M phase [8]. In gastric cancer cells (BGC-823 or SGC-7901 cells), PCB2 exerted the effects of anti-proliferation, more specifically, it stimulated apoptosis by enhancing the activities of executor caspase-3 and initiator caspase-9 and induced autophagy by increasing the two believable autophagic marker of Beclin1 and Atg5. More interestingly, PCB2 might boost both apoptosis and autophagy through Akt/mTOR pathway [81]. All pathways mentioned in this section are concluded in detail in Figure 3.

## 3. Delivery Systems of Procyanidins

However, there are some stability and bioavailability issues with the proven benefits of PCs. For example, OPC is unstable under environmental conditions, while PPC has the disadvantage of low water solubility. In order to improve the stability, bioavailability and reduce adverse effects of PPC and OPC, a variety of biodegradable encapsulation materials were used to develop a sustained-release delivery system for PPC and OPC. These drug delivery systems which were described in Table 1 make quercetin easy to be absorbed and prolong drug duration.

### 3.1. PLA and PLGA Nanoparticles

PLGA and PLA are FDA-approved biodegradable polymers that have been widely used as biomaterials for the synthesis of nanoparticles with sustained, controlled and targeted drug delivery [82]. PLGA or PLA polymers greatly improve the surface area to volume ratio of the core and the controlled release ability of nanoparticles [83]. In addition, they display a high drug loading capacity and a controlled drug release profile with improved in vivo stability for the co-delivery of various categories of anticancer agents [84].

Fernandez et al. found that PCs nanoparticles loaded with PLA have uniform spheres and smooth edges, and thermal stability increases with the improvement of PCs entrapment efficiency, suggesting that novel nanomaterials with prolonged release will enhance the potency of PCs [39]. PCs-loaded nanoparticles with the biodegradable PLGA enhance the structural stability, surface/volume mechanical and biochemical properties of demineralized dentin in collagenase-containing solutions, and resistance to biodegradation over time [40]. Although PLA/PLGA have constituted parts of a procyanidin-releasing system, most of these studies have been in vitro and need to be further expanded to complement and improve the use of PCs in vivo.

### 3.2. Polysaccharide Based Nanoparticles

Chitosan (CHT) is a polysaccharide obtained by the deacetylation of chitin and has been used as a nano-carrier for novel drug delivery systems due to its biodegradability and biocompatibility [85]. CHT has been reported to prevent the degradation of polyphenols and enhance their absorption in the gastrointestinal tract [86]. Previous studies have demonstrated that oligomeric procyanidins/bletilla striata polysaccharide/chitosan (OPC/BSP/CHT) microspheres have significant antioxidant activity compared with free OPC [41]. By encapsulating these OPC into biodegradable polymer bio-adhesive microspheres, the vulnerability of procyanidins to oxidation in air and its optical instability can be overcome and bioavailability can be further improved [42]. Furthermore, a cranberry procyanidins extract from the plant Vaccinium macrocarpon was encapsulated in CHT, resulting in increased stability, as well as molecular adhesion to extra-intestinal pathogenic *Escherichia coli* [43]. Iannone et al. synthesized chitosan microencapsulated GSP, and evaluated the pharmacological activities of GSP and CHT-containing microparticles on various cancer cells showed an increase in antitumor effect due to increased cell interaction [44]. In addition, Zou et al. prepared cacao procyanidin-gelatin-chitosan nanoparticles. Compared with PCs in solution, the stability and biological activity of PCs were significantly improved by nano encapsulation. Apoptosis of human acute monocytic leukemia THP-1 cells was observed at low concentrations [45].

**Table 1:** The physico-chemical characters of different PCs delivery vehicles.

Delivery System	Chemicals/Polymer Used	Preparation Methods	Size Range (nm)	Evaluations on the Encapsulated PCs	Effect	References
PLA nanoparticles	PLA	No data	256	Chemical stability	Sustained release of PCs from PLA nanoparticles.	[39]
PLGA nanoparticles	PLGA	Nanoprecipitation	195.4 ± 23.8	Chemical stability	The biodegradation resistance of demineralized dentin was improved by loading collagen crosslinking agent into biodegradable polymer nanoparticles via dentin tubules.	[40]
Polysaccharide-based nanoparticles	Chitosan; lauryl succinyl	Ionic gelation	458 ± 11; 3640 ± 33	In vitro cytotoxicity in HEK-293 cells	The encapsules of PCs reduce toxicity and improve chemical stability.	[86]
Polysaccharide-based nanoparticles	chitosan	Hydrogen bond	367.3–293.2	Chemical stability; the ExPEC invasion of gut epithelial cells in vitro.	PCs inhibited invasion of gut epithelial cells by ExPEC.	[44]
Polysaccharide-based nanoparticles	Gelatin; chitosan	Hydrogen bond; hydrophobic interaction and electrostatic interaction	344.7	Chemical stability; In vitro apoptotic; necrotic; and cytotoxic properties in THP-1 cells	The stability and biological activity of PCs were improved by nano encapsulation.	[45]
Protein-based nanoparticles	Poly-lactic acid	Hydrogen bond	256	Chemical stability	The encapsulation of PCs effectively enhanced the antioxidant activity	[46]
Protein-based nanoparticles	Zein	Hydrogen bond and hydrophobic interactions	392–447	Solubility; in vitro cytotoxicity in HL-60 cells	PCs-zein nanoparticles decreased the cytotoxicity of procyanidins in HL-60 cells	[47]
Modified hydroxyapatite inorganic nanoparticles	Hydroxya-patite	Metal chelation	20–50	Chemical stability	Enhanced the colloidal stability of nHAp particles	[48]
Metallic nanoparticles	Gold	Metal chelation	20–25	Chemical stability	PCs-gold nanoparticles can be used as biocompatible gold nanoparticles for medical applications; molecular imaging and cancer therapy.	[49]
Metallic nanoparticles	Gold	Metal chelation	20–40	Chemical stability	PCs-gold nanoparticles might serve as anticancer agents in killing cancer.	[50]
Metallic nanoparticles	Gold	Metal chelation	6–24	Stability and in-vitro methods employed in antidiabetic studies	PCs-gold nanoparticles have potential anti-diabetes and anti-oxidation effects.	[51]
Metallic nanoparticles	Silver; gelatin	Metal chelation	150–230	Chemical stability; antibacterial assessment; cytotoxicity test	GSP/Gelatin Composite Fibers Contained Silver Nanoparticles had the potential for applications in antimicrobial tissue engineering and wound dressing.	[52]
Metallic nanoparticles	Silver; chitosan	Metal chelation	150	Cytotoxicity patterns; the antipro-liferative activities; and the possible mechanisms of anticancer activity in HpG2 cells	The nanoparticles exhibited high anticancer activity against HpG2 cells and induced apoptosis by down-regulating Bcl2 gene and up-regulating p53.	[53]
PUDL	Ultradeformable liposomes	Thin film hydration method	140.6 ± 19	Chemical stability	PUDL could increase the transdermalflux; prolong the release and improve the stability of PCs; and could serve as an effective dermal delivery system for procyanidins.	[54]
SLN	Solid lipid	The melt-emulsion method	243	Chemical stability; evaluation of anti-oxidant activity	SLN loaded with GSP exhibit antioxidant effects for longer than free GSP.	[55]

Notes: PCs, procyanidins; GSP, grape seed procyanidins; PLGA, poly(lactic-co-glycolic acid); PLA, poly(D,L-lactic acid); ExPEC, extra-intestinal pathogenic *Escherichia coli*; Bcl-2, B-cell lymphoma-2; PUDL, ultradeformable liposomes; SLN, solid lipid nanoparticles.

### 3.3. Protein-Based Nanoparticles

Protein is biodegradable, metabolizable, symmetrical and easy to operate, which can be used to prepare nanoparticles. In recent years, some researchers synthesized whey protein-polyphenol aggregates, which not only increased the stability and shelf life of PCs, but also reduced the expression of inflammation-related genes [87]. Huang et al. synthesized tannic acid (TA)/PCs and gelatin (GLT) colloidal complexes due to spontaneous hydrogen bonding between PCs and gelatin. PCs is known to have strong antioxidant activity, and the antioxidant activity of GLT is greatly improved after complexing with polyphenols [46]. Zou et al. prepared cranberry procyanidins-zein nanoparticles using an improved liquid phase dispersion method. The oligomer with higher polymerization degree had higher loading efficiency than the oligomer with lower polymerization degree, indicating that it had greater binding affinity for zein. The results showed that hydrogen bonding and hydrophobicity were the main interactions between PCs-zein. Cell culture studies using human promyelocytic leukemia HL-60 cells showed that PCs encapsulated in nanoparticles reduced cytotoxicity compared to free PCs [47].

### 3.4. Modified Hydroxyapatite Inorganic Nanoparticles

Biomimetic hydroxyapatite (Hap, Ca_10_(PO_4_)_6_(OH)_2_) has good biocompatibility and bioactivity, which is an ideal bone replacement material and biomolecular transport matrix. Zhou et al. prepared nHAP-GSP particles with a diameter of 20–50 nm using grape polyphenols. As a biocompatibility mediated matrix, GSP can effectively regulate the nucleation and growth of hydroxyapatite nanocrystals in solution. Nucleation of nHAp crystals begins with the formation of a complex between calcium ions and phenolic hydroxyl groups in the GSP molecule. The stable aqueous dispersion of nHAP-GSP can be maintained for more than five days, which may increase its in vivo bioavailability. The strong interaction between polycarbonate and hydroxyapatite inorganic nanoparticles makes polycarbonate difficult to decompose, which is of great significance for the application of drug carrier nanocomposites with uniform organic/inorganic properties in the biomedical field [48].

### 3.5. Metal Nanoparticles

Metal nanoparticles, green synthetic silver nanoparticles (AgNPs) and gold nanoparticles (GNPs) are the most common [88]. It is notable for its unique physical properties related to distance, size and shape [89]. Catalytic gold (Au) with specific enzymatic activity is considered as an ideal choice for drug delivery [90]. The gold nanoparticles were synthesized using PCs-rich grape polyphenols, and catechins-the monomeric unit of PCs, were taken as the representative compound in the experiment. Catechins interact with the metal through chelation reactions (using special covalent bonding) to synthesize gold nanomaterials. In addition, PCs as reducing agents or stabilizers can also be used to control the size of gold nanomaterials [49]. Subsequently, another research group further explored GSP-gold nanoparticles using A431 cell line. The results showed that GSP-gold nanoparticles may be used as anticancer drugs to kill cancer [50]. Gold nanoparticles produced by chelation of PCs showed good stability in repeated centrifugation and re-dispersion experiments compared with PCs, indicating the formation of biostable and bioactive gold nanoparticles with potential anti-diabetes and anti-oxidation applications [51]. Due to the synergistic effects of PCs and special covalently bonded gold nanomaterials, it can conclude that the unique chemical structure of PCs also makes them excellent reductants and capping agents, with the ability to synthesize stable, safe and biologically active metal nanoparticles, and contribute to the medical effect [91].

Since they can interfere with bacterial metabolism, silver nanoparticles are commonly used as an antibacterial material, thereby extending the shelf life of drugs or nutritional medicines [92]. Due to these excellent properties, GSP/gelatin composite nanofiber films containing silver nanoparticles were successfully prepared by electrostatic spinning technology. Using GSP as reducing agent, AgNPs were synthesized in gelatin aqueous solution and then electrospun into nanofibers. The results showed that the synthesized fiber membranes had antibacterial properties and had potential application prospects in tissue engineering and wound dressing [52]. Another study reported green synthesis of CHT-PCs-Silver nanoparticles using chitosan/grape leaf water extract (GLE) nanoparticles as reductants and stabilizers. The cytotoxic pattern, antiproliferative and anticancer activity of the nanoparticles were investigated at the molecular level. The results showed that the nanoparticles exhibited high anticancer activity against HpG2 cells and induced apoptosis by down-regulating Bcl-2 gene and up-regulating p53 [53].

### 3.6. PUDL and SLN

Chen et al. developed a novel vesicular carrier procyanidins, namely PUDL, to expand the application range of PCs. Compared with the PCs solution, PUDL can increase the transdermal flux of PCs, prolong the release time, and improve the stability of PCs, which can be used as an effective PCs skin delivery system [54]. SLN loaded with GSP exhibit antioxidant effects for longer than free GSP, suggesting controlled release of their payload, intracellular stability and long-term persistence, and reduction of oxidative stress and inflammation [55].

## 4. Application and Treatment for Oral Diseases

### 4.1. Oral Cancer

Oral cancer is a devastating disease, often disfiguring and debilitating, and in severe cases life-threatening, including cancers of the mouth, larynx, hypopharynx and oropharynx. Head and neck cancer accounts for three percent of all cancers in the United States, and about 65,000 Americans are diagnosed with it each year [93]. Furthermore, in line with incidence, the 5-year relative survival rate for oral cancer remains a dismal 18%, and the overall mortality rate for oral and pharyngeal cancer has increased by 0.5% per year from 2009 to 2018 [94]. The causes of oral cancer are complex and include lifestyle factors such as alcohol consumption and smoking, which are strongly associated with the progression and aggressiveness of most head and neck cancers [95]. Currently, surgery, chemotherapy, and radiotherapy are the main treatment methods for oral cancer [96]. Although patients’ survival time has improved with advances in surgery, chemotherapy, radiotherapy and other treatments, drug adverse effects, pain, and drug resistance remain major problems which trouble cancer patients and physicians [97]. There are many problems with current chemotherapeutic drugs. Although these drugs can delay tumor growth and prolong survival, they are controversial in cancer treatment due to their lack of desired therapeutic effect, frequent drug resistance and strong toxic adverse effects [98,99,100].

Ongoing studies of PCs have demonstrated significant chemoprophylaxis and chemotherapy potential for oral cancer [101]. Therefore, extracting highly efficient and low toxic procyanidins from natural products to replace or combine with existing chemotherapeutic drugs may become a new research trend.

Tongue squamous cell carcinoma (TSCC) is the most common oral squamous cell carcinoma. Despite significant advances in combination therapy, five-year survival in patients with TSCC has not improved significantly, which is due to local recurrence and lymph node metastasis. Yang et al. found that GSP significantly inhibited Tca8113 cell viability and induced apoptosis in a dose-dependent manner. This was associated with the significantly increased expression of pro-apoptotic regulator Bax protein and significantly decreased expression of anti-apoptotic regulator Bcl-2 protein at 100 µg/mL GSP. Additionally, GSP significantly inhibit the secretion of matrix metalloproteinase-2 (MMP-2) and MMP-9, and Tca8113 cell proliferation, migration, invasion by inhibiting the Akt/NF-κB signaling pathway. These results indicate that GSP is expected to be a novel chemoprophylaxis agent for TSCC [102]. In addition, it has been reported that PCs can induce the apoptosis of human oral squamous cell carcinoma (HSC-2) and human salivary gland tumor (HSG), and its mechanism may be through the activation of caspase-3, caspase-9 and the degradation of cytokeratin18 to promote cell apoptosis. Meanwhile, hepatocyte growth factor (HGF) of normal gingival fibroblasts was protected [103]. GSP can inhibit the spread of oral cancer by a mechanism based on the activation of key apoptotic regulatory [101].

### 4.2. Periodontitis/Peri-Implantitis

Periodontitis is a multifactorial, multi-microbial infection, characterized by destructive inflammatory processes affecting periodontal tissues, including supporting structures such as the gums, cementum, periodontal membrane, and alveolar bone. About 5% to 15% of the world’s population is affected by a severe form of periodontitis, which can lead to tooth loss and systemic complications [104,105]. Previous studies have demonstrated that the high antibacterial and immunomodulatory activity of procyanidins makes them an interesting class of phytonutrients for the prevention and treatment of periodontal diseases [106,107].

GSP was found to reduce periodontal inflammation and alveolar bone loss by decreasing αmatrix metalloproteinase and hypoxia-inducible factor levels and increasing osteoblast activity in diabetic rats with periodontitis [108]. For the treatment of periodontitis, PCs from blueberry and cranberry have been shown to inhibit biofilm formation and the adherence of major periodontal pathogens, such as *Porphyromonas gingivalis* (*P. gingivalis*) and *Actinobacillus*, exert anti-inflammatory properties, and reinforce epithelial barrier integrity [109]. The effects of PCs or flavane-3-alcohols on the growth, colony formation, and metabolic activity of potential pathogens, as well as inhibition of pathogens’ adhesion to oral mucosal cells, have been reported in a number of studies [109]. Savickiene et al. demonstrated that PCs significantly reduced the viability of *P. gingivalis* and the non-pathogenic commensal *Streptococcus salivarius* [110]. Furthermore, PCs have greater antioxidant capacity and exhibit unique antimicrobial activity, selectively targeting Gram-negative bacteria and disease-causing strains in periodontal and peri-implant conditions, such as *P. gingivalis*, while retaining the vitality of the beneficial oral symbiotic *streptococcus salivarius* [110]. Moreover, Jekabsone et al. suggested that cranberry procyanidins inhibited the attachment of *P. gingivalis* to periodontal tissues and reduced bacterial biofilm formation, collagenase activity and invasion by neutralizing periodontopathogen proteinases and cytotoxicity, but they did not interfere with *P. gingivalis* [111]. La et al., in addition to the above activities, showed dose-dependent inhibition of *P. gingivalis* produced by type A cranberry procyanidins on the surface of dental matrix gel-coated polystyrene and inhibition of extracellular proteases from type I collagen degradation [110].

Peri-implantitis, similar to periodontitis, is an irreversible disease involving hard and soft tissue surrounding the implant, with progressive bone resorption (biological remodeling beyond bone loss), reduced bone binding, increased pocket depth, and peri-implantitis of the functional implant [112]. Due to the versatility of procyanidins, it is speculated that procyanidin-coated implant surfaces may inhibit osteoclast activity and bacterial invasion and promote healing of surrounding tissues. La et al. used cranberry procyanidins A type to inhibit the adhesion of *P. gingivalis* matrix to the surface of polystyrene and found that PCs inhibited the degradation of type I collagen by the extracellular proteases produced by *P. gingivalis* in a dose-dependent manner [113]. This provides a promising strategy for avoiding peri-implantitis after implant surface coating.

### 4.3. Dental Caries

Dental caries is a chronic progressive disease occurring in the hard tissue of teeth, which is the result of many factors, among which bacteria is the most important cause [114]. Although the prevalence of dental caries in developed countries has decreased significantly and decreases with age, it remains one of the most prevalent chronic diseases in the world [115]. PCs can effectively inhibit caries through two pathways: (1) reducing caries-causing pathogens, such as *Streptococcus mutans* (*S. mutans*) and their biofilms; and (2) promoting the mineralization of hydroxyapatite. PCs have significantly reduced the incidence of caries on smooth surfaces in animal studies, and fluoride (or fluoride in combination with PCs is more effective than PCs alone [116].

EPS produced by *S. mutans* derived GTF is an important virulence factor related to the formation of caries-causing biofilms. PCs treatment can effectively reduce the content of insoluble EPS and prevent the growth of *S. mutans* in the mixed species biofilm. As a result, the 3D structure of cranberry-treated biofilms was severely impaired, suggesting that the EPS matrix was defective and unable to form microcolonies on salivary coated hydroxyapatite (sHA) surfaces. In addition, topical application of procyanidins significantly weakened the mechanical stability of biofilms [64]. In rat caries model, local application of cranberry procyanidins during biofilm formation reduced the biomass and insoluble polysaccharide of *S. mutans* formation in vitro, and significantly reduced the incidence of caries and light caries damage [117]. The results showed that selected water extracts of potential contained high concentrations of polyphenols, such as tannins and phenolic acids, as well as caries-preventing flavonoids, since they have shown antibacterial activity against *S. mutans* in vitro and inhibit plaque formation [118].

The reconstruction of inorganic matrix is an important process of dentin re-mineralization [119]. D.J. Epasinghe et al. compared the effects of pretreatment of three flavonoids (6.5% procyanidins, quercetin and naringin) on human demineralized dentin, and found that procyanidins could improve the biomechanical properties of dentin matrix while remineralizing root caries [120,121]. Cai et al. also obtained similar results, and showed that the recovery rate of microhardness, elastic modulus and creep of demineralization dentin was significantly improved after treatment with PCs combined with silver fluoride-diamine fluoride/potassium iodide (SDF/KI) for 24 h. Compared with SDF/KI alone, minerals in caries are more evenly distributed and ions are absorbed into deeper tissues [122]. PCs therapy may lead to a new alternative or adjunct to anti-biofilm/anti-caries chemotherapy agents.

### 4.4. Endodontic Root Canal Infections

Endodontic root canal infections, divided into primary and secondary infection, is a bacteria-caused dental disease. *Enterococcus faecalis* has a low prevalence in primary root canal infections (4–40%) and a high prevalence in secondary infections (24–77%) [123]. Relationships between PCs content and antioxidant capacity, antibacterial activity against *Enterococcus faecalis* and in vitro cytotoxicity have been documented [124]. After *Enterococcus faecalis* was introduced into human dentin tubules for culture for one week, the dentin specimens were pretreated with 2%, 5%, or 10% PCs. It was found that PCs could kill *Enterococcus faecalis* in biofilm and improve the biological stability of dehydrated dentin collagen matrix. The clinical application of PCs can assist root canal rinses to play antibacterial and anti-root fracture effects [125].

### 4.5. Diseases of the Oral Mucosa

Recurrent aphthous stomatitis (RAS), also known as recurrent oral ulcers and canker ulcers, is the most common oral mucosal disease, affecting about 5–25% of the general population [126]. Procyanidins therefore have the potential to be used as a treatment for RAS. A clinical trial has shown that PCs found in grape seeds, along with other flavonoids, play an important role in the healing of skin wounds [127].

In a randomized clinical trial, 24 patients with RAS were randomly divided into drug group and placebo group to be observed the occurrence of ulcer size reduction, wound healing and pain relief. Compared with placebo group, drug group met heal of ulcer in the first 10 days of treatment, the pain relief lasted for more than 4–5 h, suggesting that the propolis extract containing PCs had a strong effect on RAS [128]. This PCs-containing oral mucosal adhesive membrane provides controlled and targeted drug delivery and may serve as a novel therapeutic strategy for the treatment of recurrent oral aphthous ulcer.

Oral candidiasis is a common oral fungal disease mainly caused by *Candida* infection [129]. It has been shown that PCA reduce the adhesion properties of *Candida* albicans by reducing inflammatory responses and interfering with NF-κB P65 activation and phosphorylation of specific intracellular kinases. PCA may help alleviate oral candidiasis by affecting the adhesion properties of *Candida* albicans and reducing the inflammatory response caused by this pathogen [130]. Further studies have shown that PCs polymeric tannins are active on *Candida* albicans biofilms and, at safe doses, not only inhibit biofilm formation and reduce the metabolic activity of mature biofilm cells, but also inhibit, at least in part, the transmission of infection mediated by this cell population [131]. PCs may be a potential drug for the prevention and treatment of oral candidiasis by affecting the virulence properties of *Candida*.

### 4.6. Dental Restoration

With respect to resin-dentin bond interface, the degradation is the primary reason for the limited durability due to the existence of hybrid layer, which caused by the hydrolysis degradation of adhesive resin and the proteolysis of collagen fiber [132]. A variety of strategies have been proposed to improve the durability of resin-dentin bonding, including the use of MMP inhibitors and collagen crosslinking agents, biomimetic remineralization, and ethanol wet bonding to improve the physical and mechanical properties of the bonding matrix (i.e., dentin) [133]. PCs are considered as collagen crosslinking agents and their effectiveness in dental collagen biomodification have been demonstrated in previous studies.

PCs can be used as a dentine base coating due to their excellent collagenous crosslinking ability. D.J. Epasinghe et al. compared the effects of three flavonoids (6.5% procyanidins, quercetin and naringin) on the properties of human dehydrated dentin, and found that elastic modulus (MOE) and ultimate tensile strength (UTS) of dehydrated dentin increased rapidly and significantly after 4 h of PCs pretreatment. The results showed that PCs could improve the biomechanical properties of dentin matrix more effectively than quercetin and naringin, and achieve better repair effect [134]. In addition, Leme-Kraus et al. again demonstrated that PCs with a higher degree of oligomerization offer a robust bioadhesion between the hydrophilic dentin matrix and the hydrophobic adhesive [135]. In addition, PCs have some antibacterial activity and are not affected by changes in concentration. Dias et al. compared the effects of the adhesives containing 2%, 4.5%, and 6% PCs and found that the addition of 4.5% PCs was beneficial to prolong the shelf life and did not affect the bonding performance. Meanwhile, no matter how high concentration of PCs was added into the adhesive, all adhesives showed similar antibacterial activity, indicating that the antibacterial effect of the adhesive was independent of the concentration of PCs [136].

Recently, Wang’s team studied methacrylate-functionated procyanidins (MAPAs), which proved that MAPAs not only overcomes the shortcomings of PCs, but also significantly improves the biological stability and crosslinking ability of dentine collagen against enzyme degradation [137]. Then, they went on to investigate the effects of MAPAs on the polymerization, microhardness, and leaching of an experimental HEMA-based dental adhesive system, demonstrating that the novel adhesive not only stabilizes dentin collagen through its PCs components, also improves the polymerization, mechanical properties and stability of HEMA adhesives through its methacrylate composition, thus leading to long-lasting dentin bonding [138].

## 5. Conclusions and Prospects

As a natural polyphenol, PCs not only have no adverse effects, but also can exert antioxidant, antibacterial, anti-inflammatory, and antineoplastic activities, and are often used as dietary supplements. By encapsulating PCs in drug delivery system including PLGA, PLA nanoparticles, polysaccharide-based nanoparticles, protein nanoparticles, modified hydroxyapatite inorganic nanoparticles, metal nanoparticles, PUDL, and SLN, we are committed to improving the bioavailability, stability, and efficacy of PCs. Currently, the application of PCs in oral diseases shows its effective treatment effect in oral cancer, such as periodontitis, dental caries and other diseases. However, only in vitro studies have been carried out, and large-scale double-blind clinical studies on PCs are needed to provide more information about their clinical efficacy and safety, so that the clinical significance of PCs as therapeutic drugs can be proposed based on sufficient scientific evidence.

## Data Availability

Not applicable.
